# The Impact of Gitelman Syndrome on Cardiovascular Disease: From Physiopathology to Clinical Management

**DOI:** 10.31083/j.rcm2308289

**Published:** 2022-08-17

**Authors:** Andrea Bezzeccheri, Gianluca Di Giovanni, Martina Belli, Rocco Mollace, Lucy Barone, Massimiliano Macrini, Alessio Di Landro, Saverio Muscoli

**Affiliations:** ^1^Department of Experimental Medicine, University of Rome “Tor Vergata'', 00133 Rome, Italy; ^2^Mediterranea Cardiocentro, 80122 Naples, Italy; ^3^Humanitas Gavazzeni, 24125 Bergamo, Italy; ^4^Division of Cardiology, Fondazione Policlinico “Tor Vergata'', 00133 Rome, Italy

**Keywords:** Gitelman syndrome, sudden cardiac death, metabolic alkalosis, hypokalemia, hypomagnesemia, hypocalciuria, arrhythmia, heart failure, hypotension

## Abstract

Gitelman syndrome (GS), or congenital hypokalemic hypomagnesemia hypocalciuria with metabolic alkalosis, is a congenital inherited tubulopathy. This tubulopathy is associated with disorders of water-electrolyte homeostasis, such as metabolic alkalosis, hypokalemia, hyponatremia, hypomagnesemia and hypocalciuria. GS has an autosomal recessive inheritance. The loss-of-function mutation involves the gene that codifies for thiazide-sensitive sodium-chloride co-transporter located in the distal convoluted tubule. The physiopathology of the syndrome is characterized by activation of the renin-angiotensin-aldosterone system (RAAS) with a low plasmatic concentration of angiotensin-II. Despite hyper-activation of RAAS, average or low blood pressure is detected in association with low peripheral resistance and reduced response to vasopressors. Clinical findings are brief episodes of fatigue, syncope, vertigo, ataxia and blurred vision; sudden cardiac death might occur. This review aims to give insight into cardiovascular implications and management of GS.

## 1. Introduction

Gitelman syndrome (GS) (OMIM 263800), also known as familial hypokalemia-hypomagnesemia or hypokalemic hypomagnesemia hypocalciuria with metabolic alkalosis [1, 2, 3], is a renal tubulopathy characterized by salt wasting leading to hypomagnesemia, hypocalciuria and secondary hyperaldosteronism responsible for hypokalemia and metabolic alkalosis [1].

The estimated prevalence is 1:40,000 [1, 3]. The disease is caused by a biallelic inactivating mutation in the SLC12A3 gene [3]. Genetic counselling helps detect carriers of putative mutations since the risk of recurrence is 25% for parents of an affected child [1, 3]. Antenatal diagnosis for GS is not advised [1, 3].

## 2. Pathophysiology

The genetic transmission of GS has an autosomal genetic inheritance which involves a mutationof the SLC12A3 gene (Solute Carrier Family 12, Member 3), which encodes the thiazide-sensitive sodium chloride co-transporter located in the apical membrane of the cell in the first segment of the distal convoluted tubule (DCT) [1, 4]. Over 350 different mutations of SLC12A3 gene have been detected [1, 5, 6, 7]. In addition, in a small percentage of GS, mutation of CLCNKB, a gene that encodes for chloride channel located in the basolateral membrane of cells of the thick ascending limb of Henle’s loop and distal convolute tubule, has been detected [1, 8]. Both these membrane channels are involved in the reabsorption of a small amount of sodium in the DCT: only 5% of sodium is usually reabsorbed in DCT.

As the effect of the loss of function of above mentioned tubular channel, volume status changes and electrolytic perturbation take place because of more sodium enters the collecting ducts as less sodium is reabsorbed in the DCT, leading to a slight volume contraction [1]. The renal loss of sodium is associated with volume depletion that activates the renin-angiotensin-aldosterone system (RAAS): the final effect of aldosterone signalling is the increase of sodium reabsorption at the collecting ducts by up-regulation of the epithelial sodium channel. Sodium reabsorption induces an electrogenic gradient that promotes potassium and hydrogen secretion. The final result is induction of hypokalemia and metabolic alkalosis [1]. The opening of calcium channel TRPV5 of the luminal side and the up-regulation of sodium-calcium co-transporter of the basal-lateral side of DCT endothelial cell increases calcium reabsorption, inducing hypocalciuria. Moreover, hypomagnesemia is one of the main features of GS. It is due to reduced magnesium absorption by lower expression of magnesium channel TRPM6 located in the DCT [1].

## 3. Clinical Manifestations

Symptoms usually appear from the age of six, with transient episodes of fatigue, tetany especially with fever or loss of magnesium due to vomiting or diarrhea, abdominal pain due to intestinal paresis, vomiting, fever, ataxia, dizziness, blurred vision, thirst, nocturia, convulsions, rhabdomyolysis, seizures, growth retardation, pubertal delay, short stature [3]. Paraesthesia, especially of the facial muscles, is common. Some patients are completely asymptomatic and GS presents as chondrocalcinosis in adolescence: swelling and brittleness of affected limbs are common [1, 3].

## 4. Diagnostic Criteria and Prognosis

The diagnosis of GS is based on clinical symptoms and typical biochemical abnormalities [1]. The criteria for suspecting a diagnosis of GS are summarized in Table 1 [3].

**Table 1.** **Criteria for suspecting a diagnosis of GS. Adapted from Blanchard *et al*. [3]**.

Chronic hypokaliemia (<3.5 mmol/L) with inappropriate renal potassium wasting (spot potassium-creatinine ratio >2.0 mmol/mmol [>18 mmol/g])
Metabolic alkalosis
Hypomagnesemia (<0.7 mmol/L [<1.7 mg/dL]) with inappropriate renal magnesium wasting (fractional excretion of magnesium >4%)
Hypocalciuria (spot calcium-creatinine ratio <0.2 mmol/mmol [<0.07 mg/mg]) in adults
High plasma renin activity or levels
Fractional excretion of chloride >0.5%
Low or normal-low blood pressure
Normal renal ultrasound

The only criteria for establishing a diagnosis of GS is the identification of biallelic inactivating mutations in SLC12A3 (sensitivity 90–100%, specificity 100%) [3]. Long term prognosis of GS is excellent [1]. However, quality of life is markedly reduced, similar to congestive heart failure (HF) or diabetes [3, 9]. Progression to chronic kidney disease is rare, although kidney disease might develop due to hypokalemia-related tubulointerstitial nephritis, tubule vacuolization, cystic changes, volume depletion and increased RAAS activity [1, 3].

## 5. Management

Asymptomatic patients affected by GS usually do not receive treatment and undergo ambulatory nephrological monitoring yearly [1].

The mainstay of treatment for GS is the recommendation for sodium-chloride intake implementation and lifelong oral potassium and magnesium supplementation [3]. Potassium- and magnesium-rich food consumption should be encouraged [3]. A suggested target for potassium may be 3.0 mmol/L and for magnesium 0.6 mmol/L (1.46 mg/dL) [3].

It is recommended dietary integration of magnesium as magnesium oxide, magnesium sulfate or magnesium-chloride with a starting dose of 300 mg/day (5 mg/kg in children) of elemental magnesium in slow-release tablets when possible; dosage titration should be based on blood levels and intestinal tolerance [1, 3]. Intravenous magnesium infusion should be reserved for patients with acute complications of hypomagnesemia such as tetany or arrhythmias (20% ${\mathrm{MgCl}}_{2}$ 0.1 mmol/kg every 6 hours) [3]. In addition, it has to be kept in mindthat if hypomagnesemia is not corrected, then potassium supplementation cannot bring potassium to normal levels [3, 10].

Potassium supplement should be given as chloride (KCl) with a starting dose of ≥40 mmol/day (1–2 mmol/kg/day in children) in 3–4 doses [3]. Intravenous KCl may be necessary in case of inability of oral intake or severe deficiency in acute settings such as arrhythmias, quadriplegia, respiratory failure, rhabdomyolysis [3]; KCl should be diluted in saline to obtain a concentration of 40 mmol/L, and 50 mmol/L should be given through a peripheral vein (maximum infusion rate 10 mmol/h) or 80 mmol/L should be given through a central venous line (maximum infusion rate 20 mmol/h) [3].

Symptomatic hypokalemia despite oral supplementation or due to low adherence or unacceptable side effects is treated by administration of potassium-sparing diuretics (mainly amiloride 5–10 mg/1.73 $m^{2}$/day; spironolactone, potassium canrenoate, eplerenone are also accepted) with monitoring of blood pressure to prevent hypotension [1, 3].

A combination of potassium-sparing diuretics, RAAS blockers and indomethacin has been proposed [3, 11, 12].

Symptoms related to chondrocalcinosis can be controlled by non-steroidal anti-inflammatory drugs; in most cases, joint surgery is not required [1, 3, 13].

Some drugs should be avoided or used with caution in patients with GS (Table 2, Ref [3]).

**Table 2.** **Drugs that should be avoided or used with caution in patients with GS due to worsening of hypokalemia and hypomagnesemia. Adapted from Blanchard *et al*. [3]**.

β_2_-receptor agonists
Insulin
Verapamil (in case of overdose)
Sodium bicarbonate
Laxatives
β-Lactam antibiotics
Aminoglycosides
Amphotericin B
Foscarnet
Loop diuretics or thiazides
Mannitol
Fludrocortisone
Topiramate
Proton pump inhibitors
Cisplatin
Calcineurin inhibitors
Mycopenolate
Anti-EGF receptors therapy
Tyrosine-kinase Inhibitors
Xanthines

Some peculiar situations should be monitored in GS. Pregnancy can worsen hypokalaemia and hypomagnesaemia, especially if situations such as vomiting or diarrhoea occur at the same time. In addition, drugs such as RAAS blockers should be discontinued because of their teratogenicity [3]. Hypokalemia and hypomagnesemia can potentiate the effects of local and general anesthetic agents; adequate blood levels of potassium and magnesium should be aimed before anesthesia [3].

## 6. Blood Pressure

GS’s clinical and hormonal features include increased plasma levels of angiotensin II (Ang II) and aldosterone due to activation of the RAAS. Though, GS patients show clinical sign such as normotension or hypotension, reduced peripheral resistance and hypo-responsiveness to vasopressors. Despite average increase of Ang II receptor number and affinity and activated RAAS, there are some evidence of Ang II signalling blockage at the post-receptor level. This has been documented by reduced gene and protein expression of the α subunit of Gq protein upon Ang II stimulation, increased of regulators G-protein signalling (RGS-2) and reduced related downstream cellular events, as protein kinase C activation by cytosolic Ca^++^ and inositol trisphosphate release [14]. Furthermore, Ang II usually increases oxidative stress via upregulation of nicotinamide adenine dinucleotide/nicotinamide adenine dinucleotide phosphate (NADH/NADPH) oxidase but in GS oxidative state is reduced. The Rho kinase downregulation in GS was associated with up-regulation of the nitric oxide (NO) system and increased NO-mediated vasodilation, partly explained by increased expression of the endothelial NO synthase (eNOS) [15, 16, 17, 18, 19, 20, 21].

Blood pressure in GS is often lower than in the general population [22]. Among relatives of patients with GS, heterozygote carriers of a SLC12A3 mutation have markedly lower blood pressure than matched controls [5]. Although the typical features of GS are normal or low values of blood pressure, some individuals may present with elevated blood pressure levels [23]. Bao *et al*. [5] performed targeted sequencing for the pathogenic gene of GS in 34 patients affected by etiologically unsolved hypertension and hypokalemia finding four variants. In GS, the short-term Ang II signalling pathway is altered by increased expression of RGS-2 protein and α-subunit of the Gq binding protein, which transduces Ang II signal: the downstream cellular events such as intracellular Ca^++^ and inositole triphosphate release and protein kinase C (PKC) activation are reduced. Moreover, downregulation of the RhoA/Rho-kinase pathway, upregulation of NO system, reduced peripheral resistance, vascular hyporeactivity, and normotension/hypotension are items that mimic the hypertensive model [14, 21]. Despite these similarities, GS is not associated with albuminuria, which correlates to reduced cardiovascular risk compared to essential hypertensive patients with albuminuria-related endothelial dysfunction [24].

The SLC12A3 gene has been investigated in a case-control study to search for single-nucleotide polymorphism (SNP) comparing hypertensive patients to normotensive subjects. There were no differences in the overall distribution of genotypes or alleles of 3 SNP (T180K, A569V, L849H) between the two groups [25].

## 7. Arrhytmias

Potassium and magnesium deficiency increases the risk of cardiac arrhythmias, which can lead to syncope or sudden cardiac death [26].

Therefore, GS has an increased risk of developing life-threatening arrhythmias due to hypokalaemia, hypomagnesaemia and prolonged QT interval, even if no significant cardiac arrhythmias were detected during the exercise test [27, 28].

Acute diarrhea or vomiting that further exacerbate hypokalemia should be treated promptly by electrolyte and fluid replacement to avoid triggering severe hypokalemia-induced arrhythmias [26].

Magnesium plays a crucial role in the active transport of calcium and potassium ions across the cell membranes, essential for maintaining vasomotor tone and normal heart rhythm. In fact, magnesium concentrations control calcium influx into cardiac myocytes during phase 0 and phase 2 of cardiac action potential, preventing intracellular calcium overload and toxicity; moreover, intracellular magnesium controls potassium shift during phase 3 that is critical in prolonging refractoriness. For this reason, GS is denoted by long QT intervals on surface electrocardiogram in up to 50% of the cases [29, 30, 31, 32]. This abnormality is related to biochemical alterations instead of an intracardial expression of the thiazide-sensitive sodium chloride cotransporter, whose gene is not expressed in heart tissue [1, 27, 33, 34]. QT interval duration seems to change marginally using the standardized potassium and magnesium supplementation [34].

Both congenital and acquired long QT syndrome (LQTS) could manifest only in salt-wasting nephropathy-associated hypokalemia [35]. In rare cases, GS causes life-threatening cardiac arrhythmias that can lead to cardiac arrest [2, 36, 37]. Tsukakoshi *et al*. [38] reported a case of a combination of GS with the SCN5A H558R polymorphism as a factor increasing the risk of developing LQTS.

On the other hand, in an observational study of patients with normal and prolonged QT interval, no rhythm or conduction abnormalities were detected in the continuous ambulatory ECG over 24 hours and in the treadmill exercise test [27].

GS patients with a prolonged QT interval should avoid participating in competitive sports [26, 39]. In addition, severe hypokalemia could induce rhabdomyolysis: the subsequent hyperkalemia and hyperphosphatemia may cause cardiac arrhythmias and acute kidney injury. For this reason, alcohol abuse, illicit drugs (cocaine, ecstasy) or commonly prescribed drugs (statins, anti-psychotic) should be used carefully in GS patients for the risk of myotoxicity [26].

An electrocardiogram should be performed at rest to assess rhythm and QT duration; further cardiology workup is indicated in case of palpitations or syncope or if the ECG abnormalities persist despite biochemical abnormalities correction [3]. In addition, an implantable defibrillator could be considered in case of relapsing malignant ventricular arrhythmia despite optimal medical therapy (potassium and magnesium supplementation, potassium-sparing diuretics, antiarrhythmic drugs) [2].

## 8. Heart Failure and Cardiac Remodelling

Ang II is one of the major contributing factors linked to cardiomyocyte hypertrophy and drug suppression of RAAS has favorable effects on left ventricular hypertrophy.

Calò *et al*. [40] studied left ventricular mass and AngII-induced ERK1/2 phosphorylation in GS, hypertensive and normotensive subjects: despite higher plasma renin activity and aldosterone in the GS group, left ventricular mass, end-diastolic volume and mass/volume ratio was significantly lower than in hypertensive patients but similar to healthy controls; the lack of cardiac remodeling in GS was associated with lower Ang II-induced ERK1/2 phosphorylation as a cellular signalling system downstream of the Ang II type 1 receptor.

The global ventricular mechanical function is directly related to the contractile properties of cardiac myocytes, which are primarily dependent on electrolyte concentration. Decreased myocardial contractility with congestive HF due to severe potassium depletion has been reported [41]. A case report demonstrated that ejection fraction was similar in hypokalemia-hypomagnesemia and normokalaemia-normomagnesemia status (after electrolyte replacement): left ventricular torsion and strain were reduced in the former status but increased after electrolyte replacement [42].

Aldosterone affects the homeostasis of sodium and potassium, blood pressure control, inflammatory reactions, cellular hypertrophy, extracellular matrix formation and apoptosis in the vessels, heart and kidney [43, 44]. Redheuil *et al*. [43] assessed the effects of aldosterone and blood pressure on myocardial fibrosis, investigating the difference in the intracellular mass index and extracellular mass index derived from cardiac magnetic resonance; intracellular mass index increased in response to increased afterload. The extracellular mass index was similar in GS, healthy individuals and hypertensive patients, while it was higher in primary hyperaldosteronism despite blood pressure adjustment: it suggests a permissive effect of high blood pressure on the pro-fibrotic effect of aldosterone.

GS is associated with reduced capacity to adapt left ventricular function in response to acute overload imposed by isometric exercise, despite normal cardiac function, dimension and perfusion at rest. During exercise, a reduced microvascular reserve has been demonstrated as a reduction of myocardial flow velocity at contrast echocardiography. In addition, the upregulation of the NO system, which causes maximal vasodilatation, is an addictive factor that limits an increase in myocardial blood flow with raising metabolic demand [39].

Dilated cardiomyopathy in GS patients has been anecdotally reported [45].

Magnesium is an essential co-factor for cellular respiration and adenosine triphosphate synthesis in mitochondria, and abnormal magnesium levels might alter the energy production of cardiac myocytes. In addition, intracellular magnesium also mobilizes calcium into the sarcoplasmic reticulum, where excitation-contraction coupling occurs, thereby modulating cardiac contraction. Moreover, magnesium can also suppress plasma aldosterone secretion, decreasing sodium and water retention. As such, hypomagnesemia might potentially increase the risk of development and progression of HF [46, 47, 48].

The main studies about cardiovascular implications of GS are summarized in Table 3 (Ref. [14, 21, 23, 24, 25, 27, 33, 35, 38, 39, 40, 41, 43]).

**Table 3.** **Main studies on cardiovascular implications of Gitelman Syndrome**.

Study ID	Study design	Population	Topic	Results	Conclusions
Calò LA [14]	Mini-review	-	Ang II signaling	-	Blockade of Ang II signalling decreased the expression of the Gq protein upon Ang II stimulation, increased the number of regulators of G protein signalling and reduced the activation of protein kinase C
			G proteins		
			Regulators of G-protein signaling and NOS		
Pagnin *et al*. [21]	NR	9 (1 BS, 8 GS)	ROK gene	Reduction	The study confirms BS/GS as a human model to investigate interrelated systems involved in the pathophysiology of hypertension and throws more light on the cellular mechanisms of BS/GS reduced Ang II short- and long-term signaling pathways
			Protein expression	Reduction	
			PAI-1 gene	Normal	
Ogihara *et al*. [23]	Case report	1	Hypertension in middle-aged woman	-	This case demonstrates that hypertension could result in spite of the extremely decreased Na reabsorption in GS and that essential hypertension is genetically heterogeneous, and abnormality of all genes may not be necessarily required to cause BP rise
Calò *et al*. [24]	Research letter	12 (2 BS, 10 GS)	UACR	Normal	UACR in BS/GS did not differ from HS, contrary to hypertensive patients, in which increased UACR reflects endothelial dysfunction and its associated increased CV risk
Aoi *et al*. [25]	Case control	315 (EH patients)	SNP of SLC12A3 gene	No difference of genotypes or alleles of any of the SNP between EH and NT groups	The casual gene of GS is not involved in determining BP levels
Foglia *et al*. [27]	NR	21	QT interval	Prolonged in 11 patients, normal in 10 patients	The results of continuous ambulatory ECG exercise testing are reassuring. Arrhythmias may occur patients with very severe hypokalaemia, during medication that prolong the QT interval or for non-adherence to the recommended regimen of care
			Continous 24-h ambulatory ECG	No arrhythmias	
			Exercise testing	No myocardial ischaemia	
			Echocardiography	No myocardial abnormalities	
Bettinelli *et al*. [33]	NR	27	QT interval	Prolonged in 11, normal in 16 patients	The corrected QT interval is often pathologically prolonged in patients with Gitelman disease, suggesting that there is an increased risk for development of dangerous arrhythmias
			Plasma K, Mg and ionized calcium	No differences in prolonged or normal QT interval	
			Plasma Na and chloride	Lower in prolonged than in normal QT interval	
			${\mathrm{HCO3}}^{-}$	Higher in prolonged than in normal QT interval	
Darbar *et al*. [35]	Case Report	1	QT interval, Plasma K, Mg		GS could be a second distinct congenital disorder modified the clinical presentation of LQTS
			Syncopal episodes		
Tsukakoshi *et al*. [38]	Case Report	1	QT interval		The SCN5A polymorphism and GS-related electrolyte disturbance may contribute to the persistent QT prolongation in some patients
			SCN5A H558R polymorphism		
Scognamiglio *et al*. [39]	NR	20	QT interval		Reduced capacity to adapt left ventricular function in response to acute overload imposed by isometric exercise, despite normal cardiac function, dimension and perfusion at rest
			Two-dimensional and myocardial contrast echocardiography		
Calò *et al*. [40]	NR	12 (2 BS, 10 GS)	Echocardiography		The lack of cardiac remodeling in GS was associated with lower Ang II-induced ERK1/2 phosphorylation as a cellular signalling system downstream of the Ang II type 1 receptor
			PRA and Plasma aldosterone		
			Ang II-induced ERK 1/2 phosphorylation		
Potts *et al*. [41]	Case Report	1	Plasma K, PRA,		
			Right and left heart catheterization		
			Kidney Biopsy		
Redheuil *et al*. [43]	NR	80	Plasma aldosterone		It suggests a permissive effect of high blood pressure on the profibrotic effect of aldosterone
			Blood pressure		
			Echocardiography		
			Cardiac Magnetic Resonance		

BS, Bartter Syndrome; GS, Gitelman Syndrome; ROK, Rho kinase; PAI-1,plasminogen activator inhibitor-1; NR, not reported; NOS nitric oxide system; Ang II Angiotensin II; BP, blood pressure; UACR ,urinary albumin/creatinine ratio; HS healthy subjects; Na, sodium; EH, essential hypertension; SNP, single nucleotide polymorphisms; NT, normotensive; K, plasma potassium; Mg, magnesium; HCO3, bicarbonate; ECG, electrocardiography; PRA, Plasma renin activity; ERK, extracellular signal-regulated kinase.

## 9. Discussion

GS is a normotensive hypokalemic tubulopathy characterized by a genetically determined functional defect of renal transporters and ion channels. GS leads to a clinical setting of hypokalemia, sodium depletion, the simultaneous occurrence of hypomagnesaemia and hypocalciuria and hyperactivation of the RAAS.

This tubulopathy is considered a benign disease, and the diagnosis is usually incidental.

**Fig. 1.**
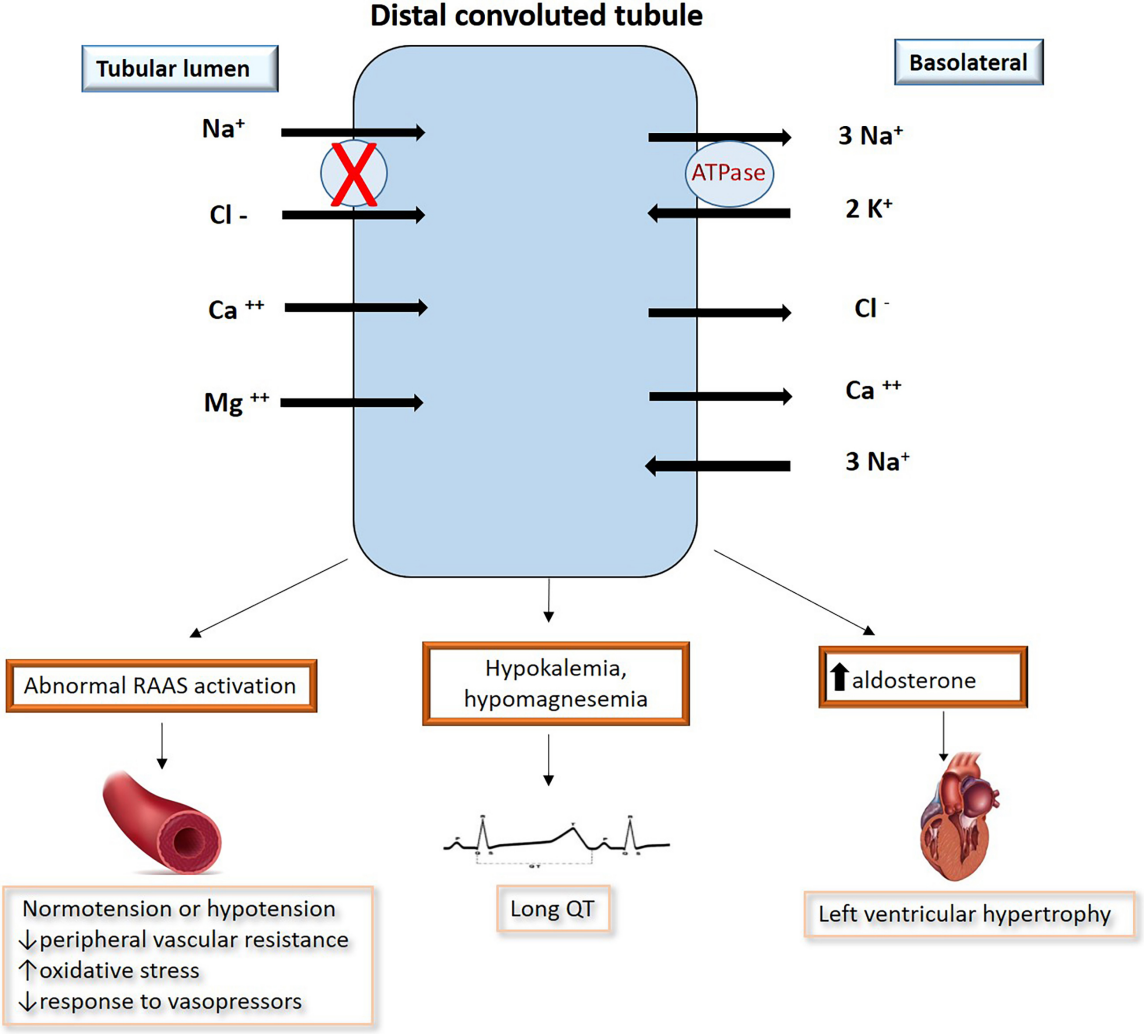
**The clinical and hormonal environment of Gitelman syndrome includes increased plasma levels of angiotensin II (Ang II) and aldosterone, activation of the RAAS in normotension or hypotension, decreased peripheral resistance and hyporesensitivity to pressor agents**. Potassium and magnesium deficiencies lead to an increased risk of developing cardiac arrhythmias that can lead to syncope or sudden cardiac death. Ang II is one of the major factor in ventricular hypertrophy.

However, the prolonged QT interval in the electrocardiogram associated with severe hypokalemia has been indicated as a pathogenetic electrophysiological mechanism underlying dangerous arrhythmias that can lead to sudden cardiac death (Fig. 1).

The prevalence of these syndromes is challenging to assess because most patients remain asymptomatic; however, these could contribute to the significant rate of unexplained sudden cardiac deaths in the general population [49].

Given the pathogenetic mechanism involved, GS could apparently drop into special population subsets of patients with prolongation of QT interval at ECG from an epidemiological point of view.

Although rare, there are some reports of patients with GS who suffered sudden aborted cardiac death [36]. In this clinical setting, it has been shown that some triggers are required to precipitate malignant ventricular arrhythmias, which can occur during exercise; patients underwent resting and isometric stress echocardiography, electrophysiological study, and coronary angiography after the interrupted SCD episode. Ventricular arrhythmias were not inducible during the electrophysiological study and coronary vessels were normal on angiography. Left ventricular dysfunction was also detected during exercise associated with the decreased cardiac index, paradoxical QTc interval prolongation and QTc interval prolongation during nocturnal vagal pacing. In addition to hypokalemia, the latter could be identified as possible additional triggers of SCD in these patients, concluding that hypokalemia may not be the only factor precipitating SCD in GS.

Therefore, isolated hypokalemia is often not sufficient to precipitate life-threatening arrhythmias but requires the presence of triggers. A systematic screening/treatment protocol for recognizing such factors is usually not performed in these patients and a structurally normal heart has been described using standard cardiac assessment. Diagnostic approaches reveal the complexity of these syndromes with several specific abnormalities of cardiac function that are largely independent of hypokalemia, including the inability to recruit myocardial contractility, causing exercise-induced left ventricular dysfunction [9, 10].

Therefore, cardiac manifestations in these syndromes may not be fully explained by potassium and magnesium deficiency. An essential role in the induction of these cardiac manifestations could be played by the altered Ang II signaling and by the upregulation of the NO system, which determines several alterations in the regulation of vascular tone in GS, reducing the microvascular reserve and consequently producing defects of myocardial perfusion, especially during exercise causing an overload of the left ventricle [14, 50, 51].

During isometric exercise, an inadequate increase in myocardial blood flow was detected due to a reduction in myocardial flow velocity, related to coronary epicardial vessel resistance and myocardial plateau intensity.

Inadequate increase in myocardial blood flow, vasodilation induced by nitric oxide and reduced perfusion pressure could be the three pathophysiological processes that limit the increase in cardiac perfusion in response to the increase in metabolic demands. The above mentioned alterations in myocardial perfusion during physical exertion can reduce the recruitment of myocardiocytes, an inability to match the acute overload of the left ventricle, and a reduction in the cardiac index. The paradoxical prolongation of the QT interval that was found during exercise in these patients could be the consequence of the interdependence between the anomalies of myocardial perfusion and the reduction of the cardiac index. The role of microvascular dysfunction and the consequent anomalies of myocardial perfusion as triggers underlying malignant ventricular arrhythmias in the context of chronic hypokalemia usually present in patients with GS would therefore indicate multifactorial pathogenesis. In this scenario, both the complexity of the anomalies in the function of the ionic channels typical of the disease and the altered intracellular signaling that lead to vascular tone dysregulation contributes to the development of ventricular arrhythmias that predispose patients with GS to sudden cardiac death.

Patients with GS have elevated levels of Angiotensin-converting enxyme-2 (ACE-2) and Angiotensin 1-7. ACE and ACE2 produce Ang II, a molecule with vasopressor properties involved in cardiovascular remodeling; on the other hand, Ang 1-7 is a vasodilator which has an anti-reshaping action on heart tissue. Although Ang 1-7 has antiarrhythmic properties, it can induce ventricular tachycardia and sudden death at higher concentrations. Therefore, ACE-2 may play an essential role in blood pressure homeostasis in the long-term complications of hypertension, such as cardiovascular remodeling and the induction of cardiac electrical abnormalities. Therefore, the demonstration of myocardial perfusion abnormalities in patients with GS arises from clinical impact as a predicting factor of an increased risk of cardiovascular mortality. Non-invasive imaging could be helpful in prognostic stratification in this regard. Myocardial perfusion imaging using single-photon emission tomography, rubidium-82 positron emission tomography and myocardial contrast echocardiography, have been used as means to define high-risk subjects among patients with various diseases and showed good prognostic value [52, 53, 54, 55, 56, 57, 58]. Electrolyte disturbances along with other cardiovascular disorders may therefore include patients with GS in a special population at higher risk of sudden death, further studies are needed to better understand the issue. GS should be distinguished from other diseases such as KICA syndrome (Kidney tubulopathy and Cardiomyopathy) [59] and Gitelman-like syndrome which are unrelated to SLC12A3 mutations [60]. KICA syndrome is a novel inherited disease caused by activating the mTOR pathway. It is characterised by tubulopathy with renal salt wasting, severe hypomagnesaemia and nephrocalcinosis, combined with dilated Cardiomyopathy. Therefore, the diagnosis of KICA syndrome should be considered in individuals with either early-onset dilated Cardiomyopathy or hypomagnesaemia of renal origin [59]. Recently, Trepiccione F. *et al*. [60] described a rare form of Gitelman-like syndrome related to a mutation of the SLC26A4 gene, which codes for the anion exchange protein pendrin. In this disease, pendrin mutations can be associated with GS-like manifestations, even in the absence of thyroid or inner ear impairments typical of Pendred syndrome. However, as this is are novel syndrome, and little is known about cardiovascular involvement, further studies are needed to understand the disease better.

## 10. Conclusions

Gitelman syndrome is a benign tubulopathy characterized by alterations in electrolytic balance such as hypokalemia, hypomagnesemia, hypocalciuria, and sodium depletion. Although GS prevalence is underestimated because of challenging and mostly incidental diagnosis, the clinical implication could be severe as heart failure, arrhythmias, and sudden cardiac death may occur. Correctly identifying the disease and what it implies is essential for optimal management to optimize outcomes.

## Abbreviations

ACE, angiotensin-converting enzyme; Ang II, Angiotensin II; DCT, Distal convoluted tubule; GS, Gitelman syndrome; eNOS, endothelial nitric oxide synthase; HF, Heart Failure; KICA, Kidney tubulopathy and Cardiomyopathy; LQTS, long QT syndrome; NADH/NADPH, Nicotinamide Adenine Dinucleotide/Nicotinamide Adenine Dinucleotide Phosphate; NO, Nitric Oxide; RAAS, Renin-Angiotensin-Aldosterone System; RGS, Regulator of G-protein signaling; SNP, Single-Nucleotide Polymorphism; SCD, sudden cardiac death.
